# Pressurized intraperitoneal aerosol chemotherapy (PIPAC) for peritoneal malignancy: initial experience of the first program in the Baltic countries

**DOI:** 10.1186/s12957-021-02357-5

**Published:** 2021-08-10

**Authors:** Rokas Račkauskas, Augustinas Baušys, Martynas Lukšta, Jonas Jurgaitis, Marius Paškonis, Kęstutis Strupas

**Affiliations:** 1 Clinic of Gastroenterology, Nephrourology, and Surgery, Institute of Clinical Medicine, Faculty of Medicine, Vilnius University, Vilnius, Lithuania; 2 Department of Surgery, University hospital of Klaipeda, Klaipeda, Lithuania

## Abstract

**Background:**

Peritoneal malignancies include primary and metastatic cancer of the peritoneal cavity. The most common origin for peritoneal metastasis is ovarian, gastric, and colorectal cancers. Irrespective of the origin, peritoneal metastases represent the advanced disease and are associated with poor long-term outcomes. The minimally invasive approach of pressurized intraperitoneal aerosol chemotherapy (PIPAC) allows repeated applications and objective assessment of tumor response by comparing histological samples. This study aimed to investigate the initial experience with PIPAC in the Baltic region.

**Methods:**

All patients who underwent PIPAC at Vilnius University Hospital Santaros Klinikos between 2015 and 2020 were included in this retrospective study. The primary outcome of the study was overall survival (OS) in patients with peritoneal carcinomatosis treated by PIPAC. The secondary outcomes included postoperative morbidity; peritoneal carcinomatosis index (PCI) and ascites reduction after treatment by PIPAC.

**Results:**

In total, 15 patients underwent 34 PIPAC procedures. PIPAC-related intraoperative and postoperative morbidity occurred in 3 (8.8%) of 34 procedures. Following PIPAC, the median PCI decreased from 8 (4; 15) to 5 (1; 16) in GC patients, although, the difference failed for significance, *p* = 0.581. In OC patients, PCI after PIPAC remained stable. Median overall survival after PIPAC procedure was 25 (95% CI 5–44) months. Ovarian cancer patients (22; 95% CI 12–44 months) had significantly higher OS, compared to gastric cancer patients (8; 95% CI 4–16 months), *p* = 0.018.

**Conclusions:**

PIPAC is safe and feasible for patients with gastric and ovarian cancers peritoneal metastases.

## Background

Peritoneal malignancies include primary and metastatic cancer of the peritoneal cavity. The most common origin for peritoneal metastasis is ovarian, gastric, and colorectal cancers [1]. Irrespective of the origin, peritoneal metastases represent the advanced disease and are associated with poor long-term outcomes [2]. Currently, systemic palliative chemotherapy remains the standard treatment for these patients, although the efficacy of such treatment is very limited. One of the limiting factors is the plasma-peritoneal barrier, which restricts the movement of the systemic chemotherapeutic drug to reach the target in the peritoneum [3]. To overcome this issue, the intraperitoneal application of chemotherapy was proposed [4]. Further, intraperitoneal chemotherapy is associated with reduced toxicity because of lower systemic concentrations [4]. Considering these advantages, hyperthermic intraperitoneal chemotherapy (HIPEC), usually combined with cytoreductive surgery, gained attention for peritoneal malignancies. Although, a series of recent studies (PRODIGE7, COLOPEC, CYTO-CHIP, PROFILOCHIP) failed to demonstrate the oncological benefit of the HIPEC [5–8]. Another available strategy for intraperitoneal chemotherapy application is pressurized intraperitoneal aerosol chemotherapy (PIPAC). The rationale behind PIPAC includes (1) optimization of drug distribution by applying an aerosol rather than a liquid solution; (2) applying increased intraperitoneal hydrostatic pressure to increase drug penetration to the target; and (3) limiting blood outflow during drug application [9, 10]. The minimally invasive approach of PIPAC allows repeated applications and objective assessment of tumor response by comparing histological samples [10, 11]. However, PIPAC remains an experimental treatment option for patients with peritoneal malignancy. Thus, this study aimed to investigate the initial experience with PIPAC in the Baltic region.

## Materials and methods

### Ethics

Vilnius Regional Biomedical Research Ethics Committee approval (No. 2020/11-1279-761) was obtained before this study was conducted. The waiver of informed consent was given by the authority. The study was conducted according to the Declaration of Helsinki.

### Patients and data collection

All patients who underwent Pressurized Intraperitoneal Aerosol Chemotherapy (PIPAC) at Vilnius University Hospital Santaros Klinikos between 2015 and 2020 were included in this retrospective study.

Data on patient characteristics were extracted from the prospectively collected institutional electronic database. They included clinicopathologic characteristics (age; gender; history of previous cancer treatment; origin, number, and size of metastases; peritoneal carcinomatosis index (PCI) score at every PIPAC procedure) and treatment-related characteristics (length of surgery; blood loss; chemotherapeutic drugs; postoperative complications by Clavien-Dindo classification).

### Technique of procedure

Indications for the PIPAC procedure were peritoneal carcinomatosis ± refractory ascites. Potentially eligible patients willing to receive experimental treatment by PIPAC were discussed at multidisciplinary team meetings and the decision for such treatment was individual in every case.

The procedures were performed following the protocol adjusted to our infrastructure [12].

All operations were performed under general anesthesia; antibiotic prophylaxis with a single dose of cefazoline 1.0 g IV was administered at the time of induction of anesthesia. A nasogastric tube and urinary drainage were not used unless there was a specific indication for their use.

After insufflation of a 12 mmHg CO_2_ open access capnoperitoneum was made, two balloon trocars measuring 5 and 10 mm were inserted into the abdominal wall. The preferred sites of insertion were the supraumbilical incision and the left iliac fossa along the same line.

An evaluation of the PCI was done. Biopsies were performed from four different regions of the peritoneal cavity, and ascitic fluid was completely drained and sent for cytological examination.

The 9-mm microinjection pump was connected to an intravenous high-pressure injector and inserted into the abdomen through the 10-mm access port.

A 5-mm camera was inserted through the other port keeping the tip of the Capnopen in view. A safety checklist was performed before the procedure ensuring there is no gas leakage.

One hundred fifty milliliters of NaCl 0.9% containing cisplatin 7.5 mg/m^2^ body surface and doxorubicin 1.5 mg/m^2^ body surface area was injected through the Capnopen at a pressure of 200 psi at the rate of 0.5 ml/s to generate the aerosol. The intraabdominal pressure throughout the procedure was maintained at 12 mmHg [12].

The therapeutic capnoperitoneum was then maintained for 30 min. Then, the chemotherapy aerosol was evacuated via a separate hospital air-waste system. Finally, trocars were retracted and laparoscopy was ended.

Patients were allowed oral liquids on the same day and discharged on the following day in the absence of adverse events.

Following procedures were repeated at 6 weeks intervals.

### Study outcomes

The primary outcome of the study was overall survival (OS) in patients with peritoneal carcinomatosis treated by PIPAC. OS was defined as the time from the first PIPAC procedure to death. The secondary outcomes included postoperative morbidity; PCI and ascites reduction after treatment by PIPAC. Data on survival and date of death were collected from the Lithuanian National Cancer Registry.

### Statistical analysis

All statistical analyses were conducted using the statistical program SPSS 24.0 (SPSS, Chicago, IL, USA). Continuous variables are presented as median with an interquartile range. Categorical variables are shown as proportions. Continuous variables were compared by a Mann-Whitney *U* test, and categorical variables by the Pearson’s chi-square or Fisher exact test, as appropriate. Related samples were compared by Wilcoxon signed-rank test or McNemar test, as appropriate. Overall rates were analyzed by the Kaplan–Meier method and compared by the log-rank test. Statistical significance was considered when a *p* value < 0.05 was achieved.

## Results

### Baseline characteristics

In total, 15 patients underwent 34 PIPAC procedures. The baseline clinicopathologic characteristics are shown in Table 1. All patients received systemic chemotherapy before PIPAC. Different regimens were used for ovarian cancer (OC) and gastric cancer (GC) patients. All OC patients (6/6; 100%) received platinum-based systemic chemotherapy, specifically paclitaxel, and carboplatin. In GC groups, patients received different schemes including XELOX, EOX, FOLFIRI, and FLOT.

**Table 1:** Baseline clinicopathologic characteristics of patients who received PIPAC

Malignancy; *n* (%)	Gastric cancer	9 (60.0%)
	Ovarian cancer	6 (40%)
Median PCI score (Q1; Q3);	Before PIPAC	8 (4; 15)
	After PIPAC	5 (1; 16)
Sex; *n* (%)	Female (*n*; %)	11 (73.3%)
	Male (*n*; %)	4 (26.7%)
Median age (Q1; Q3); years		58 (51; 68)
Median hospitalization (Q1; Q3); days		5 (3; 6)
Median BMI (Q1; Q3)		25 (20; 30)
History of radical surgery for primary tumor; (*n*; %)	Yes	8 (53.3%)
	No	7 (46.7%)
Median CA125 level (Q1; Q3); kIU/l		103 (15; 351)
Median CEA level (Q1; Q3); ng/l		1.4 (0.5; 9.6)
Median CA19.9 level (Q1; Q3); ng/l		12.3 (6.9; 75.9)
Number of PIPAC procedures	1	5 (33.3%)
	2	2 (13.3%)
	3–4	8 (53.4%)
Median operation time (Q1; Q3); min minutes		115 (110; 133)

### PIPAC procedure characteristics

**Fig. 1:**
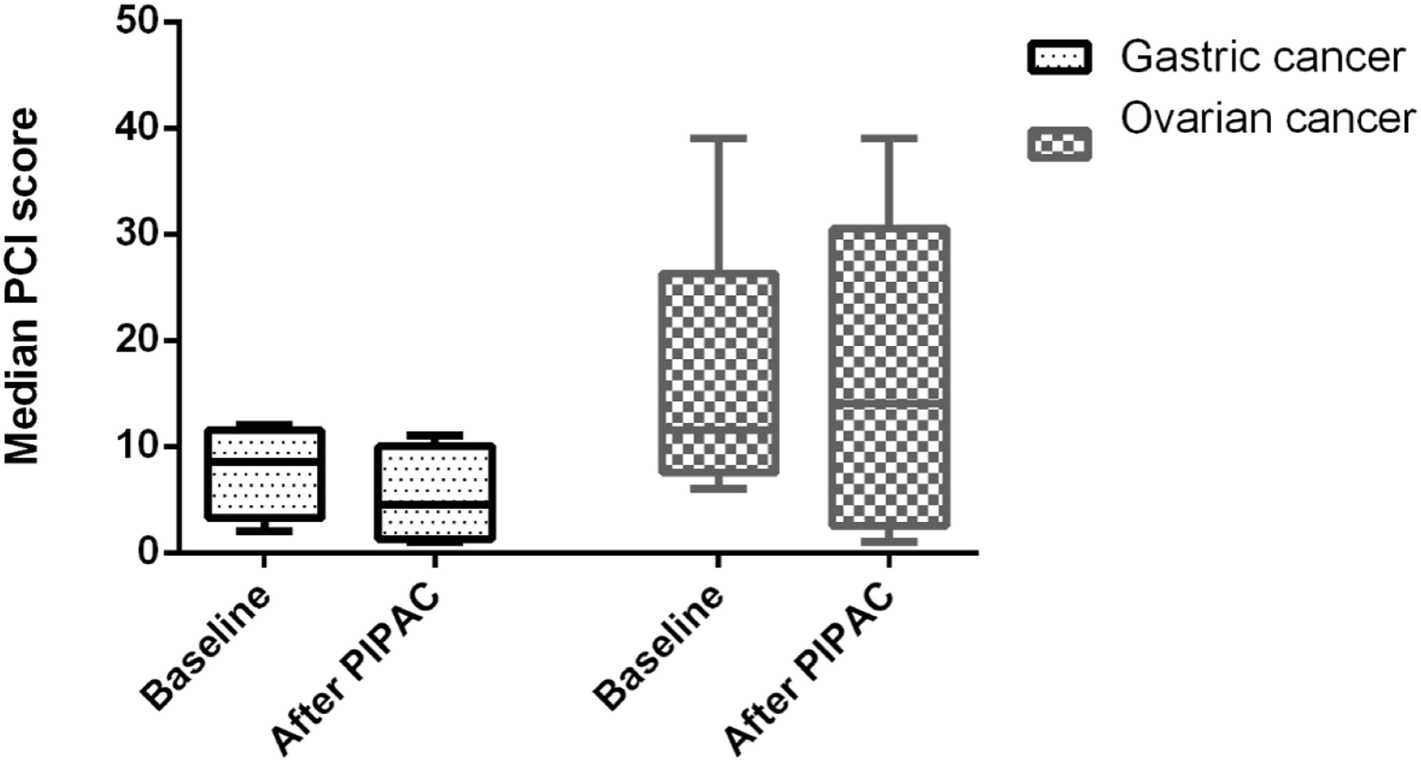
Median peritoneal carcinomatosis index in patients who received PIPAC for gastric and ovarian cancer peritoneal metastases

One, two, or three and more PIPAC procedures were performed for 5 (33.3%), 2 (13.3%), and 8 (53.4%) patients, respectively. Following PIPAC, the median PCI decreased from 8 (4; 15) to 5 (1; 16), although, the difference failed for significance, *p* = 0.999.PIPAC stabilized the PCI score in both—patients with GC and OC (Fig. 1). One of the indications for palliative PIPAC is refractory ascites. Among 10 patients who received at least 2 PIPACs, 7 had ascites at baseline with a median volume of 300 ml (Q1 100; Q3 2200). After PIPAC, 2 (28.6%) of these patients had no ascites and the median volume decreased to 50 ml (Q1 35; Q3 4050); however, the difference was not significant, *p* = 0.500. PIPAC-related intraoperative and postoperative morbidity occurred in 3 (8.8%) of 34 procedures. One patient developed severe postoperative neutropenia (2.8%) after PIPAC (Clavien-Dindo score 2); one patient (2.8%) developed intraabdominal abscess postoperatively, which was managed with ultrasound drainage (Clavien-Dindo score 3a); and in one case (2.8%) bowel was perforated during initial port placement due to extensive adhesions, it was repaired intraoperatively, and patient’s further recovery was uneventful.

### Long-term outcomes

**Fig. 2:**
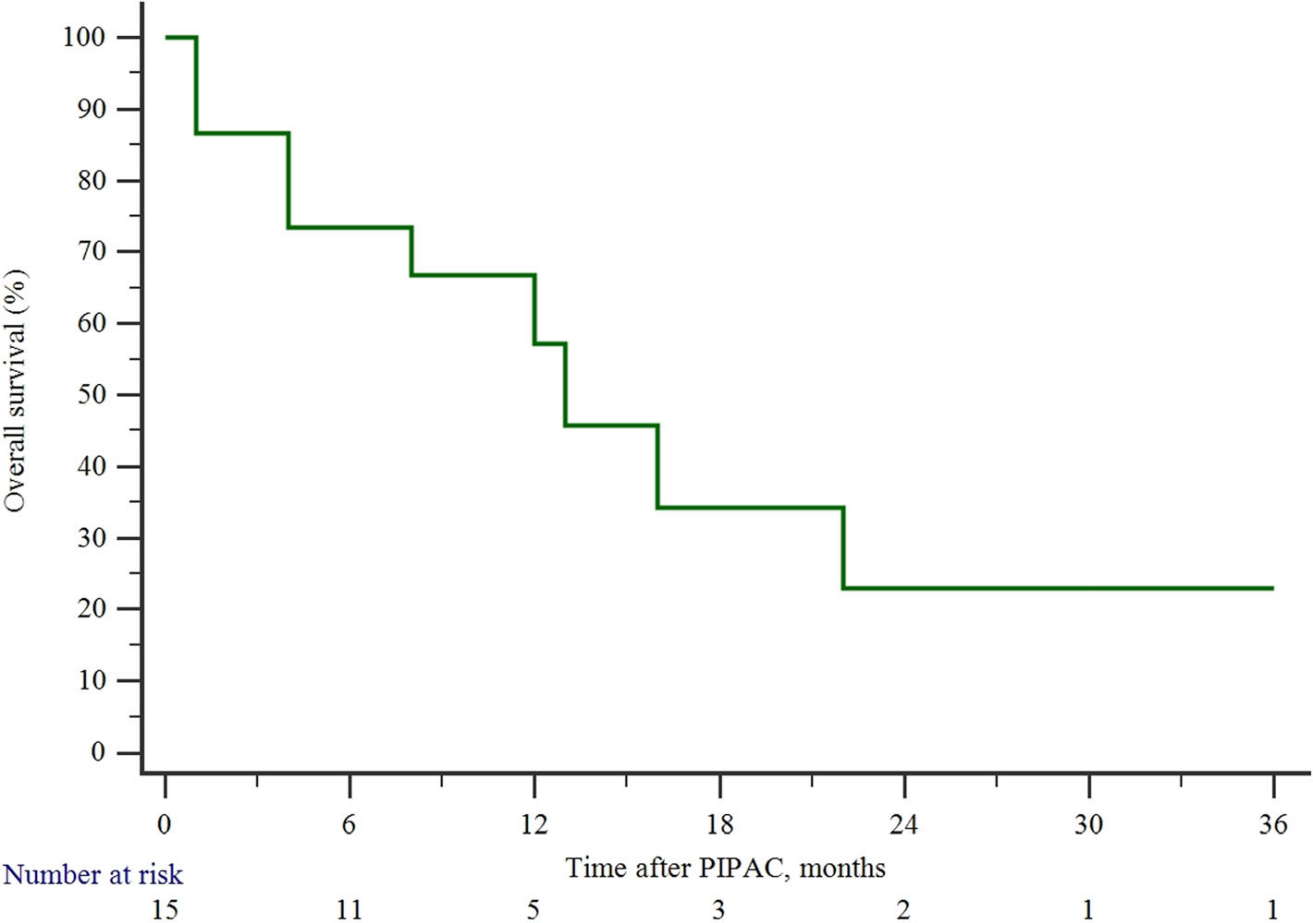
Overall survival in the entire cohort of study patients who received PIPAC for peritoneal metastases

**Fig. 3:**
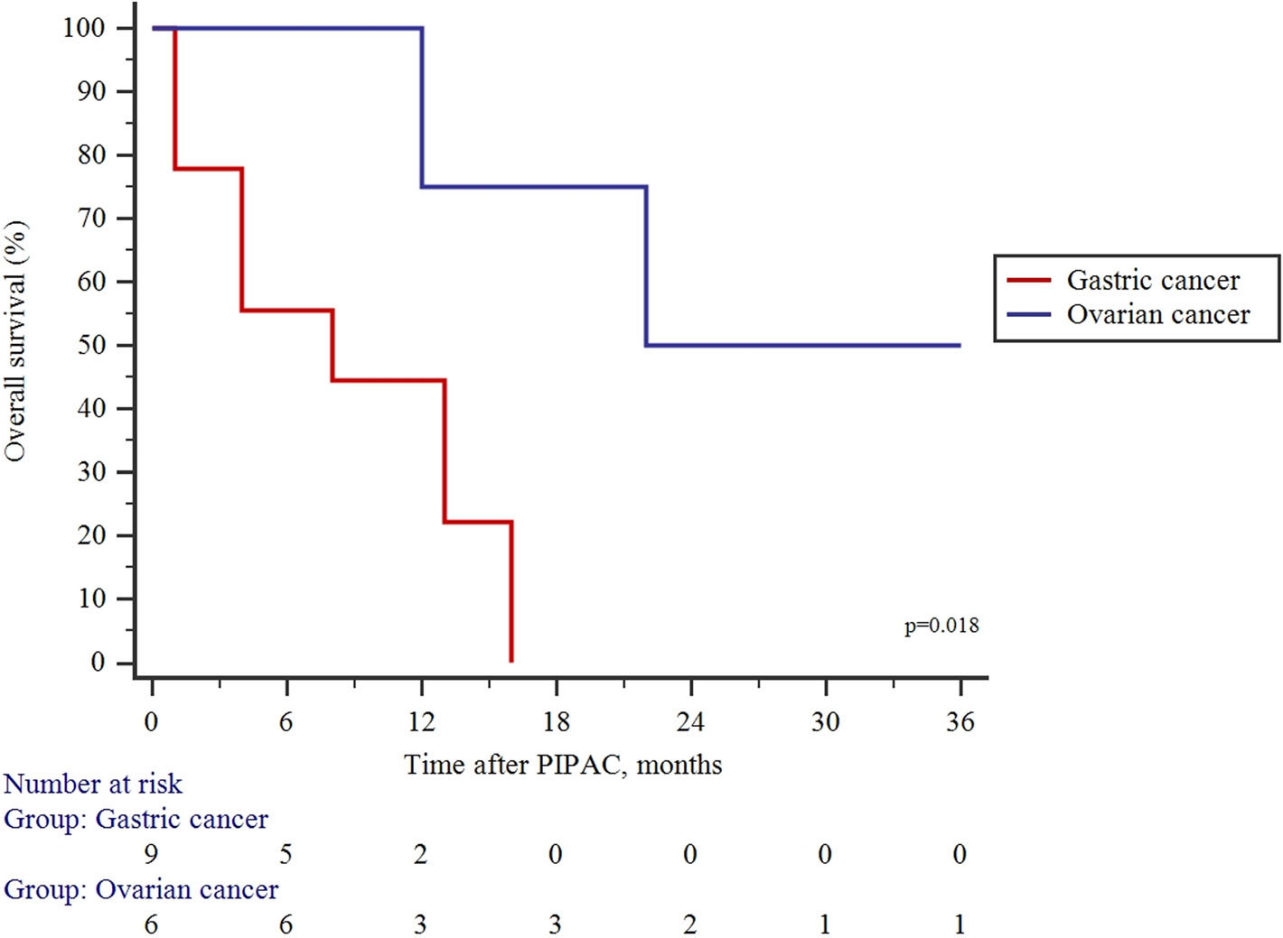
Comparison of overall survival between gastric and ovarian cancer patients treated by PIPAC

The median time to follow-up after PIPAC was 10 (Q1:4; Q3: 16) months and the median survival by Kaplan–Meier analysis was 25 (95% CI 5–44) months (Fig. 2). OC patients (22; 95% CI 12–44 months) had significantly higher OScompared to GC patients (8; 95% CI 4–16 months), *p* = 0.018 (Fig. 3).

## Discussion

The present study demonstrated the initial results of the first PIPAC program in the Baltic region country—Lithuania. PIPAC was safe and feasible for patients with gastric and ovarian cancer peritoneal metastases. The repeated PIPAC procedures were performed for 66.7% of patients, and postoperative complications occurred after 8.8% of procedures, with no postoperative mortality. PIPAC reduced the mean PCI in gastric cancer patients and stabilized the disease for ovarian cancer patients.

PIPAC is a new and emerging technique for peritoneal metastases of various cancers. Some evidence shows that it is one of the best methods to manage the burden of advanced intraperitoneal cancer by reducing or halting disease progression and improving quality of life [13]. Further, the minimally invasive approach is one of the major advantages of the PIPAC procedure, as it is associated with a low intraoperative and postoperative morbidity ranging between 0 and 11% in previous and our study [1]. A typical candidate for PIPAC suffers from miliary peritoneal carcinomatosis, which is considered an incurable disease. Although, PIPAC can stabilize the progression of peritoneal carcinomatosis and sometimes even downgrade the disease to the level, where potentially curative cytoreductive surgery with or without HIPEC is feasible [14]. In the present study, we found stabilization of the disease in ovarian cancer patients and regression of the PCI in gastric cancer patients, although the difference failed for significance. GC patients with a limited PCI may benefit from curative cytoreductive surgery + HIPEC as shown by a recent meta-analysis [15]. Thus, because of PCI score reduction after PIPAC in GC patients, it may be considered as a conversion therapy from unresectable to potentially resectable disease.

A second most common indication for PIPAC is a refractory accumulation of ascites, which impairs quality of life [16]. It has been reported that PIPAC is an excellent method to control ascites, thus it improves the quality of life at the final stages of the disease [17]. In our study, we have found that 28.6% of patients suffering ascites resolved after PIPAC. Further, the median volume of ascites decreased substantially, although the difference failed for significance.

The highest effect of PIPAC is achieved when procedures can be repeated. Alyami et al. reported a clinical response rate of 50–90%, in cases where 3 PIPACs were utilized [1]. Our results show that repeated PIPAC procedures are feasible in approximately two-thirds of patients. Although, the utilization of repeated PIPACs depends on the origin of peritoneal metastases, as three cycles were feasible for 83.3% with OC and only one-third of patients with GC. Such differences may be explained by the different severity of the disease by different origin peritoneal metastases [1]. The different origins of metastases are also, associated with different prognoses. Grass et al. reported that median survival following PIPAC ranges between 11–14.1 and 13.4–15.4 months, for OC and GC patients, respectively [11]. In contrast, our study demonstrated a longer survival for OC patients. The unclarities on the subgroup of patients who benefit the most from PIPAC have to be elucidated in future clinical studies.

A minimally invasive approach associated with low morbidity and potential therapeutic effect for incurable disease makes PIPAC an attractive novel treatment strategy for peritoneal metastases. Thus, there is a growing number of clinical studies investigating PIPAC for various types of cancers and various combinations with systemic therapy or even PIPAC as neoadjuvant therapy [17–21]. Furthermore, some novel anti-tumorigenic agents, such as taurolidine are under investigation for PIPAC [22]. These novel agents may increase the effectiveness and thus the attractiveness of PIPAC. Although to date, there is a lack of robust evidence from prospective randomized studies on the efficacy of PIPAC, thus it still has to be considered as an experimental treatment option.

Our study has some limitations. The retrospective design and small sample size are the major limitations that could lead to the selection bias and underestimation of the positive and negative effects of PIPAC for gastric and ovarian cancer patients with peritoneal metastases. Therefore, the findings of the current study must be validated with larger cohorts.

## Conclusions

The present study demonstrated the initial results of the first PIPAC program in the Baltic region country—Lithuania. PIPAC was safe and feasible for patients with gastric and ovarian cancer peritoneal metastases.

## Data Availability

The datasets used and/or analyzed during the current study available from the corresponding author on reasonable request.

## Abbreviations

OC: Ovarian cancer
GC: Gastric cancer
PIPAC: Pressurized intraperitoneal aerosol chemotherapy
PCI: Peritoneal carcinomatosis index
OS: Overall survival
HIPEC: Hyperthermic intraperitoneal chemotherapy
